# Public Willingness to Pay for and Participate in Sanitation Infrastructure Improvement in Western China's Rural Areas

**DOI:** 10.3389/fpubh.2021.788922

**Published:** 2022-01-06

**Authors:** Simei Wu, Yang Zhang, Bao-Jie He

**Affiliations:** ^1^School of Management, Xi'an University of Architecture and Technology, Xi'an, China; ^2^Centre for Climate-Resilient and Low-Carbon Cities, School of Architecture and Urban Planning, Chongqing University, Chongqing, China; ^3^Key Laboratory of New Technology for Construction of Cities in Mountain Area, Ministry of Education, Chongqing University, Chongqing, China

**Keywords:** sustainable development, rural area, willingness to pay, willingness to participate, sanitation improvement, motivations

## Abstract

The suitability and feasibility of public-private partnership (PPP) patterns in a rural context have not been well-documented and understood. To address this research gap and practical plight, this study aims to analyze the rural resident's willingness to pay for and participate in the improvement of rural sanitation facilities, and further explore the drivers and barriers affecting their decisions. This study was performed in rural areas of three western provinces, including Shaanxi, Ningxia, and Inner Mongolia, of western China's rural areas by conducting a survey on 1,248 rural residents. In Inner Mongolia, the proportion of respondents who were willing to pay was highest, while the proportion of respondents who may provide labor was lowest among the three provinces. Respondents from Ningxia had the least willing to pay, and respondents from Shaanxi had the highest willingness to participate. Overall, respondents' rural (living) duration time, personal interest in local government notice, and the latest time when the sanitation facilities were improved could significantly affect their willingness. In Inner Mongolia, occupation and water availability could significantly influence respondents' willingness, and both gender and health conditions had significant impacts. In Ningxia, respondents' personal interest in local government notice had a notable impact on willingness, and low-income respondents showed a more notable willingness to pay and participate. In Shaanxi, occupation and water availability could significantly influence respondents' willingness. Respondents' personal interest in local government notice had a notable impact on their willingness. This study is of significant importance to understand rural resident's participation in sanitation infrastructure improvement to support relevant PPP projects, and is important to solve poverty-caused dilemmas.

## Introduction

Poor sanitation quality has been a critical problem for many countries, with severe impacts on underground water quality, public health, and well-being. It is more prominent in poor and rural areas of developing countries because of the lack of sanitation infrastructure and services. Such a problem impedes the achievement of the Sustainable Development Goals in aspects of *No poverty, Good health and well-being, Clear water and sanitation, Industry, innovation and infrastructure, Reduced inequalities*, and *Sustainable cities and communities*. A report from the World Health Organization asserted that 9% of the rural population worldwide has sewer connections. In the least developed countries, 56% of the population does not even have limited sanitation services, and a larger part of this population are rural residents (1). Existing studies have reported that inadequacy of sanitation is the cause of diseases, leading to about 502,000 deaths annually (2–4). The absence of sanitation could further bring damages to underground water and public health (5). Moreover, sanitation is also one of the drivers to rural-urban inequality, where there was an alarming situation in the sanitation facilities in India, and the rural-urban inequality damaged sustainable human development (6). It is further pointed out that inadequate maintenance of sanitation facilities imposes a considerable burden on the resilience of cities (7). Sanitation, therefore, is regarded as the fundament of creating employment and reducing poverty (8). Groundwater pollution and health threats are caused by poor sanitation facilities in China's vast rural areas (9). Although accompanied by economic development, the Chinese government has also made great efforts to upgrade sanitation facilities. Because of the imbalance in regional development, sanitation facilities in western China are still inadequate (10). In western China, there is an urban-rural gap in the development of sanitation (11). As a result, western rural China is a developing region in urgent need of sanitation upgrades.

The improvement of rural sanitation has been an urgent task, and governments in both developed and developing countries have made multiple attempts. However, the demand for funds to improve rural sanitation is so high that government finances usually cannot fully support the cost. As a result, the progress of improving sanitation in rural areas is slow globally. Many local governments, fortunately, are seeking solutions to such problems. For instance, the government of Peru improved sanitation after approving the funding support from the United States Agency for International Development, and technical support from the Centers for Disease Control and Prevention United states (12). In the slums of Kisumu, Kenya, a nongovernment organization (NGO) initiated a sustainable holistic sanitation project. Through capacity-building and the creation of organizational structures, the Dutch NGO improved local sanitation with limited funding (13). In Tanzania, the government received support from the World Health Organization and United Nations Children's Emergency Funds to implement the improvement of sanitation technologies (14). In Mandalay, Myanmar, the local government introduced constituency funds and large-scale international funds to upgrade sanitation. Under multilateral donors, the government used technologies to obtain the city's resilience (15).

Nevertheless, a donation is not the solution to sanitation problems. Because of limitations in national budgets and funds, many local governments have to collect funds by themselves. The PPP approach has been considered an efficient means to procure infrastructure and has been widely adopted in various national projects in developed and developing countries (16–19). In particular, the PPP mode has been invited by the water sector to reduce the economic pressures of desalination and drainage projects in Saudi Arabia (20). In Ghana, the government tried to use PPP to support public investment priorities and develop water infrastructure (21). In Sweden, the PPP project was used in large and complex projects to support the development of water infrastructures (water and sewage) in cities (22). Existing literature has further concluded that the success of PPP schemes is affected by the following critical factors: (i) financial: stability of the economic environment, the profitability of the project, cost effectiveness and financial attractiveness, reliable contractual arrangements, project preparation, and resource availability; (ii) political: stability of the political environment, government guarantee on the sound financial package, transparent regulatory framework, the experience of government in PPP schemes; (iii) technical: project complexity, reliable private sector with great technical strength and experience; and (iv) Social: long-term demand of service need, and public and community support (23–27).

Overall, the PPP mode provides an innovative and practical way for local governments to upgrade rural sanitation. Rural governments can potentially raise a part of the funds from rural residents, and, alternatively, residents can possibly cooperate with local governments to build sanitation facilities in their villages by performing a part of the labor during construction. Accordingly, improvement in sanitation could be more efficient and cheaper. However, attitudes of local residents toward payment and participation, namely, willingness to pay (WTP) and willingness to participate (WTPP), are critical questions for understanding (28–31). However, there are only few studies focused on PPP in rural areas. Harvey et al. (32) found the PPP schemes effectiveness promotes sanitation development in 155 rural communities of Uganda. Josphat and Kimathi (33) focused on a PPP project funded by the Dutch government to upgrade sanitation technologies in rural Kenya and found that enthusiasm facilitators could significantly accelerate the implementation of the PPP project. Josphat et al. (34) further suggested that PPP schemes could provide financial inclusion, having a positive impact on market and demand, and resulting in the promotion of the development of sanitation in rural Kenya.

However, the attitude of rural residents has merely been study in the majority of these literature. The literature on the attitude of rural residents who participated in the PPP program is limited to be able to show progress and sanitation improvements. The mechanism behind rural residents' WTP and WTP in sanitation improvement is still unknown, especially when rural residents are generally poor. To build effective and appropriate sanitation facilities in rural areas of China, therefore, it is essential to focus on the attitude of rural residents on PPP programs to upgrade sanitation. Accordingly, this article aims to assess rural residents' WTP for improving sanitation and their WTPP by providing labor during improvements. Furthermore, this article will explore factors that can impact rural residents' willingness to improve sanitation by improving sewage treatment equipment and collection systems. Using surveys and binary logistical regression, we examined the factors affecting subjects' willingness with the PPP approach in Ningxia, Inner Mongolia, and Shaanxi. The results of this study could support residents to improve rural infrastructure to solve a poverty-caused dilemma and improve the quality of their lives. It could further provide critical information and insights for poor countries and regions.

## Study Area

The study was conducted in rural areas of three provinces, namely, Ningxia, Shaanxi, and Inner Mongolia, in the western part of China. The three provinces have a large rural population and they suffer water shortage problems because of their uniform climate and elevation (35, 36). As a result of drought, the local ecology is fragile and difficult to repair, and wastewater can bring lasting impacts on the local ecology. To make matters worse, the water shortage is aggravated by the absence of sanitation (37). In 2016, the total sewage discharge in Inner Mongolia had reached 1.05 billion tons/year. The municipal sewage treatment capacity of Inner Mongolia was 2.44 million m3/day in 2019 (38). By 2016, the total sewage discharge in Ningxia had reached.34 billion tons/year. The municipal sewage treatment capacity of Ningxia was 1.09 million m3/day in 2019 (39). By 2016, the total sewage discharge in Shaanxi had reached 1.67 billion tons/year. The municipal sewage treatment capacity of Shaanxi was 3.97 million m3/day in 2019 (40). According to these data, the sewage treatment capacity of the three provinces cannot meet their own needs. To make matters worse, the wastewater produced in many rural areas is not counted in the total sewage discharge in the provinces. A report from NBOs asserts that only 15% or less of villages could have proper sewage treatment (41), and that more than 85% are under challenges. Therefore, sanitation in these areas need to be improved urgently.

Local governments of the three provinces expressed a strong interest in improving rural sanitation and implemented the “Beautiful Countryside” policy for improving the environment, roads, water, sanitation, and toilets. The local governments prioritized sanitation because the construction of sanitation facilities in these provinces is far behind other types of infrastructure, and poor sanitation poses terrible hazards to public health and the environment. Inner Mongolia contains the largest pastoral area of China. In Inner Mongolia, 38% of the population resides in rural areas, 76% of the population is aged between 15 and 64 years, and less than 5% of the population is illiterate. The economy of Inner Mongolia is dominated by cattle production and tourism. In 2019, the per capita disposable income was USD 4,430, and per capita consumption expenditure was USD 3,008 in Inner Mongolia (42). Ningxia, with a rural population of 3 million, where rural residents account for 42% of the population in this region, 73% of the population is aged between 15 and 64, and 7% of the population is illiterate. The economy of Ningxia is dominated by agriculture and tourism. The per capita disposable income was USD 3,540, and per capita consumption expenditure was USD 2,653 in Ningxia in 2019 (42). Shaanxi has a rural population of 17 million. It is the cultural and scientific research center of west China. In Shaanxi, 73% of the population is aged between 15 and 64 years, less than 6% of the population is illiterate, and 43% of the population are rural residents. Shaanxi's economy is dominated by agriculture and tourism. The per capita disposable income was USD3,577, and per capita consumption expenditure was USD 2,532 in 2019 (42).

## Research Method

### Questionnaire

The characteristics of local residents and sanitation were investigated in the survey. The survey questionnaire included four main sections based on a contingent valuation method (CVM) (43, 44). The first section focused on the rural residents' socioeconomic characteristics, the second considered personal activity characteristics, the third was about the characteristics of services and infrastructure, and the fourth focused on rural residents' WTP and WTPP, affordable cost, and the main reason for being willing or unwilling to pay.

The questionnaire focused on the payment for sanitation improvement in building a collection system and purchasing miniature sewage treatment equipment. Labor included installing miniature sewage treatment equipment and building a collection system in a respondent's house and villages. Costs of improving sanitation did not include the cost of maintenance and operations. The open-ended approach was simple but rarely resulted in usable responses, so our approach allowed objections to avoid selection bias (45).

It should be noted that there was no ethics committee working particularly for questionnaire approval in our affiliations. However, during the questionnaire design, to respect the privacy of possible respondents, we invited eight experts to test the questionnaire if the questions were appropriate. Moreover, to conduct and understand group reactions to particular problems for an accurate survey (46), we invited four target groups with 20 participants in total to explore the questionnaire design and assess the income and affordability level. Each target group, consisting of five members, was comprised of local rural residents, government officials, and academic experts, and the duration of each group discussion was 1.5 h. Based on the opinions and suggestions generated from the four target group discussions, the questionnaire was revised and upgraded. To guarantee the WTP bid range for the CVM and validate the survey, the questionnaire was pre-tested on 30 rural residents and amended based on feedback. Overall, the group discussion and pre-test allowed for the questionnaire to be well understood by rural residents.

**Figure 1:**
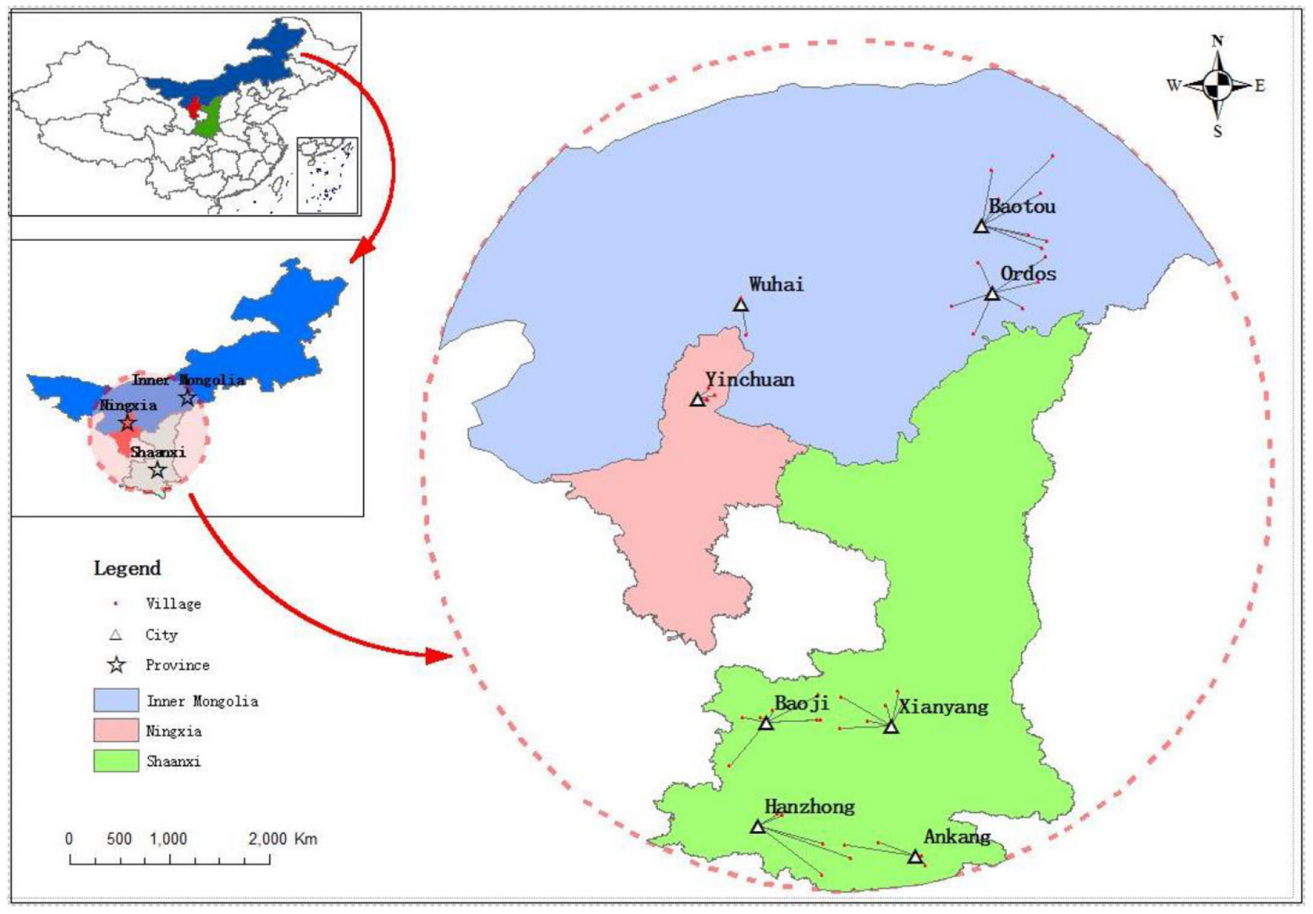
Study area.

The study was conducted from May to December 2019, and stratified random sampling was adopted. According to the population size of each province, one city from Ningxia, three cities from Inner Mongolia, and four cities from Shaanxi were chosen for the survey. According to the population size of each city, four villages from Yinchuan, six villages from the Ordos, seven villages from Baotou, two villages from Wuhai, seven villages from Xianyang, six villages from Baoji, five villages from Hanzhong, and four villages from Ankang were chosen for the survey. The level of economic development of the survey villages varies, as does the condition of sanitation. Generally, sanitation facilities in the survey villages need to be upgraded. Overall, the survey villages are a good representation of the condition of sanitation in local rural areas. The spatial distribution of the study area is shown in Figure 1.

In each village, we randomly selected 10% of the population. During the questionnaire survey, the interviewees were informed that they could quit the survey at any time if they felt uncomfortable and that the results would be anonymous and only for research. This procedure guaranteed that the questionnaire study was conducted on a voluntary basis. The respondents were encouraged to ask questions and request clarification during the survey. If the respondents were illiterate, we provided the questionnaires orally. We received 1,402 questionnaires, and 154 were excluded, as they were incomplete or contradictory. Finally, the response rate was 89%, with 1,248 valid questionnaires.

### Model Description

Logistic regression has been widely used in studies that focus on WTP and WTPP (47–49). Since the dependent variable was a two-category variable in the WTP and WTPP in this study, binary logistic regression was adopted to explore the relationship between the dependent variable and influential factors (50, 51). The two possible outcomes are represented by response variable 1 if the respondent is willing to pay or participate, and 0 if the respondent is unwilling to pay or participate. We estimated binary logistical regressions of the following type (50–52):


(1) $$\begin{array}{l} {\text{P}}_{n}\left(i\right)=\frac{e^{\left(\hat{\beta }\right)}}{1+e^{\left(\hat{\beta }\right)}}\text{ where }\hat{\beta }=\beta_{0}+\beta_{1}{\text{X}}_{1,n}+...+\beta_{i}{\text{X}}_{i,n}, \end{array}$$

where P_*n*_(*i*) is the probability that a rural resident is willing to pay or participate, $\hat{\beta }$ is a vector of estimated parameters, and X is a vector of explanatory variables used to determine the outcome probability of P_*n*_(*i*) being equal to 1. For each group, an alpha of 0.05 was used to screen the significance of variables. Odds ratios (ORs) were computed for the variables, and a confidence interval of 95% was used.

## Results

### Socioeconomic, Pollution, and Sanitation Characteristics

Table 1 presents the demographic characteristics of the respondents. There were more female respondents than male respondents in both Inner Mongolia and Ningxia, while there were more male respondents in Shaanxi. About 8.05–17.65% of the respondents were unhealthy, following an increasing order of Shaanxi, Inner Mongolia, and Ningxia. Regarding education level, 21.76–25.74% of the respondents received high school education without large differences among the three provinces, while Ningxia had the highest proportion of University education receivers (36.76%), followed by Inner Mongolia (16.33%), and Shaanxi (9.24%). Additionally, Ningxia had the highest proportion of farmers (50.74%), Shaanxi had the highest proportion of service personnel (32.04%) and self-employed (46.05%), and Inner Mongolia had the highest proportion of migrant workers (22.68%). Only a minority of respondents' family size was larger than six in all the provinces (10.29–26.38%). In Shaanxi, 43.67% of the respondents' annual household income was lower than USD 1,510. The annual household income of 66.21% of the respondents from Inner Mongolia and 69.85% of the respondents from Ningxia was higher than USD 4,532.

**Table 1:** Descriptive statistics of demographic characteristics.

**Variables**	**Group**	**Inner Mongolia**	**Ningxia**	**Shaanxi**
		**(*N =* 441)**	**(*N =* 136)**	**(*N =* 671)**
Gender	Female	235(53.29%)	70(51.47%)	297(44.26%)
	Male	206(46.71%)	66(48.53%)	374(55.74%)
Health status	Unhealthy	60(13.61%)	24(17.65%)	54(8.05%)
	Healthy	381(86.39%)	112(82.35%)	617(91.95%)
Education	Primary school	140(31.75%)	10(7.35%)	136(20.27%)
	Secondary school	129(29.25%)	41(30.15%)	327(48.73%)
	High school	100(22.68%)	35(25.74%)	146(21.76%)
	University and above	72(16.33%)	50(36.76%)	62(9.24%)
Occupation	Farmer	108(24.49%)	69(50.74%)	55(8.20%)
	Service personnel	67(15.19%)	3(2.21%)	215(32.04%)
	Migrant worker	100(22.68%)	25(18.38%)	92(13.71%)
	Other work	166(37.64%)	39(28.68%)	309(46.05%)
Family size	≤ 6	383(86.85%)	122(89.71%)	494(73.62%)
	>6	58(13.15%)	14(10.29%)	177(26.38%)
Annual	<1510 USD	60(13.61%)	22(16.18%)	293(43.67%)
household	1510–4532USD	89(20.18%)	19(13.97%)	192(28.61%)
income	>4532USD	292(66.21%)	95(69.85%)	186(27.72%)

*All exchange rates to USD are 6.62 because the data were collected in 2018*.

Table 2 presents the personal activities of the respondents. Regarding time spent in the villages, the majority of the respondents (52.94–76.60%) could spend over 10 months in the villages annually. However, about 42.65% of the Ningxia respondents only lived in the villages for less than 3 months annually, and the proportion in Inner Mongolia was about 27.21%. About 9.24–22.06% of the respondents thought they frequently checked the notices of village government. Learning policy of upgrading sanitation was a common activity among 57.37%, 46.32%, and 80.03% of the Inner Mongolia, Ningxia, and Shaanxi respondents, respectively. However, more respondents (53.68%) in Ningxia were reluctant to learn the policies.

**Table 2:** Descriptive statistics of personal activity characteristics.

**Variables**	**Group**	**Inner Mongolia (*N =* 441)**	**Ningxia (*N =* 136)**	**Shaan'xi (*N =* 671)**
Time spent in village (each year)	Less than 3 months	120(27.21%)	58(42.65%)	46(6.86%)
	4–9 months	34(7.71%)	6(4.41%)	111(16.54%)
	10–12 months	287(65.08%)	72(52.94%)	514(76.60%)
Notices checking (Frequency of checking the village	Least frequent	152(34.47%)	40(29.41%)	280(41.73%)
government's notices)	Less frequent	128(29.02%)	44(32.35%)	123(18.33%)
	Moderately frequent	86(19.5%)	22(16.18%)	206(30.70%)
	Frequent	46(10.43%)	17(12.50%)	39(5.81%)
	Most frequent	29(6.58%)	13(9.56%)	23(3.43%)
Learning policy (Upgrading sanitation)	No	188(42.63%)	73(53.68%)	134(19.97%)
	Yes	253(57.37%)	63(46.32%)	537(80.03%)

Table 3 presents the relevant services, infrastructure, and willingness characteristics. About 11.76–25.78% of the respondents had suffered different levels of difficulties in getting safe drinking water, following a decreasing order from Shaanxi (25.78%), Inner Mongolia (19.50%), and Ningxia (11.76%). Most of the sanitation facilities have not been upgraded in the past 5 years, especially in Inner Mongolia (78%) and Ningxia (62.5%). About 21.76% of the sanitation facilities have not been upgraded in the last 10 years in Shaanxi. Most of the toilets had not been connected with sanitation facilities to treat feces (91.84% in Inner Mongolia, 91.91% in Ningxia, and 188.97% in Shaanxi). About 30.15–57.82% of the respondents were willing to pay for sanitation, with the highest proportion in Inner Mongolia (57.82%), followed by Shaanxi (50.67%), and lowest in Ningxia (30.15%). About 66.74–70.96% of the respondents were willing to provide labor to improve sanitation, with the highest proportion in Shaanxi (74.96%), followed by Ningxia (66.44%), and Inner Mongolia (66.91%). Overall, the respondents in Inner Mongolia were the most positive on WTP, while the respondents in Shaanxi were the most positive on WTPP. The lowest proportion of WTP of Ningxia may be due to Ningxia having the lowest per capita disposable income and the second highest per capita consumption expenditure among the three provinces. The residents in Ningxia are more nervous about their money than those in the other two provinces. As a result, the Ningxia residents are more sensitive to spending money.

**Table 3:** Descriptive statistics of services, infrastructure, and willingness.

**Variables**	**Group**	**Inner Mongolia (*N =* 441)**	**Ningxia (*N =* 136)**	**Shaanxi (*N =* 671)**
Difficulties of water (Difficulties to get safe drinking water)	No	355(80.5%)	120(88.24%)	498(74.22%)
	Yes	86(19.5%)	16(11.76%)	173(25.78%)
Time of upgraded (The last time of sanitation upgraded)	≥10 years	263(59.64%)	45(33.09%)	146(21.76%)
	5–10years	81(18.37%)	40(29.41%)	160(23.85%)
	≤ 5years	97(22.00%)	51(37.50%)	365(54.40%)
Connected toilets (Toilets connected with sanitation)	No	405(91.84%)	125(91.91%)	597(88.97%)
	Yes	36(8.16%)	11(8.09%)	74(11.03%)
Willing to pay (WTP)	No	186(42.18%)	95(69.85%)	331(49.33%)
	Yes	255(57.82%)	41(30.15%)	340(50.67%)
Willing to provide labor (WTPP)	No	148(33.56%)	45(33.09%)	168(25.04%)
	Yes	293(66.44%)	91(66.91%)	503(74.96%)
Affordable cost for willing rural residents (per household)	38 USD	173(68%)	24(59%)	291(86%)
	76 USD	54(21%)	12(29%)	43(13%)
	113 USD	18(7%)	3(7%)	3(1%)
	153 USD	10(4%)	2(5%)	3(1%)

**Figure 2:**
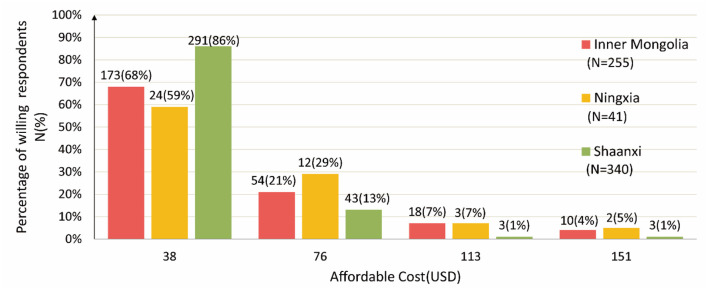
Fixed-amount quota and proportion of rural household payment.

Figure 2 shows the details of the affordable cost of rural respondents (willing) in three provinces. Overall, the proportion of affordable cost decreased with an increase in expenditure in all the three provinces. Namely, the respondents' WTP for sanitation improvement would decline as payment increased. This finding is consistent with the economic theory of demand (53). Furthermore, the results indicate that the mean fixed payment of rural residents was USD 55.75 in Inner Mongolia, USD 59.82 in Ningxia, and USD 45.2 in Shaanxi per household.

### Factors Shaping Attitudes

**Figure 3:**
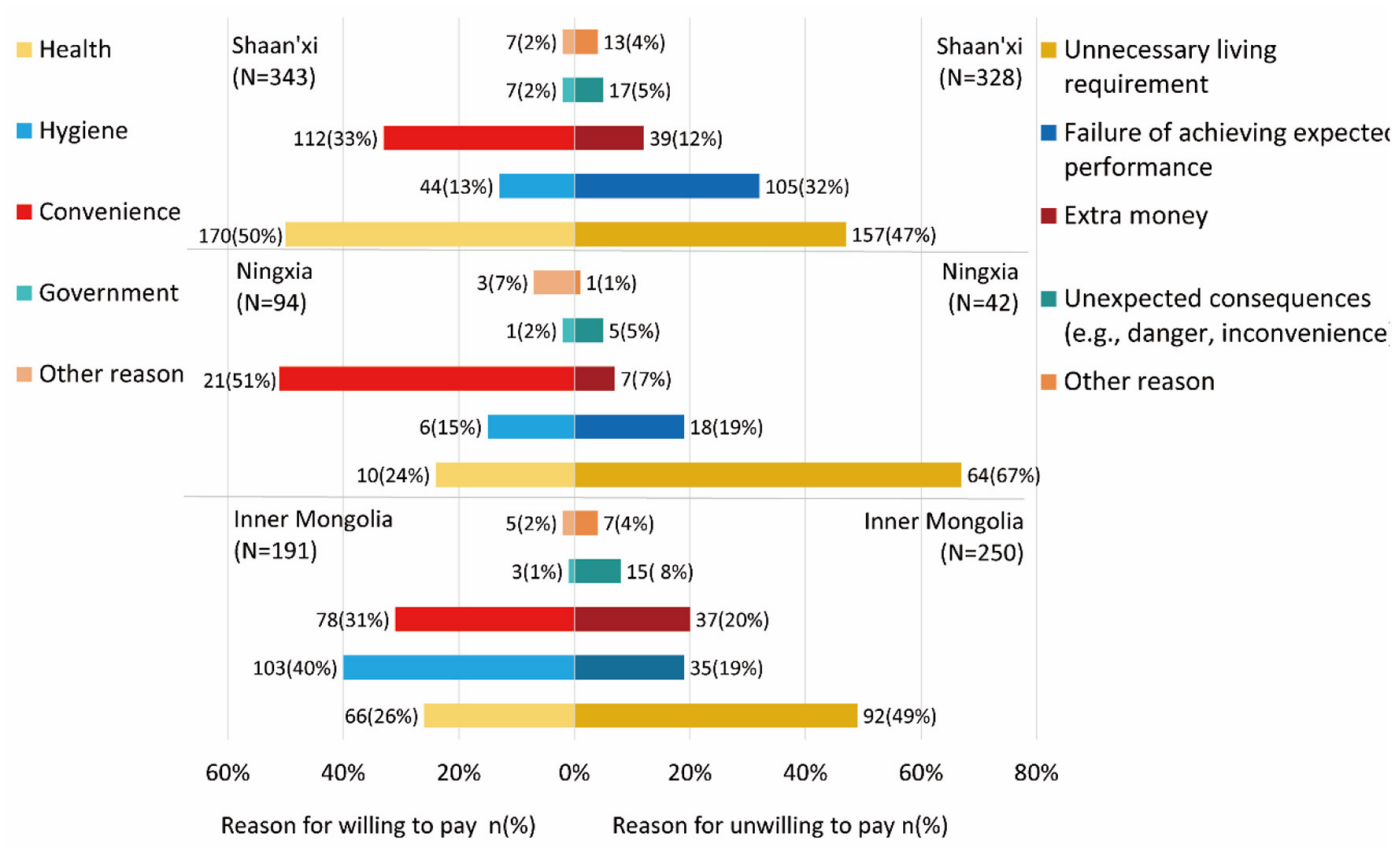
Reasons for the choices.

Motivations behind respondents' decisions on WTP and WTPP were further investigated, as shown in Figure 3. In Inner Mongolia, the main motivations to pay were hygiene (40%), convenience (31%), and health (26%). In addition, only 1% of the respondents who were willing to pay indicated their support for the government policy, and other motivations accounted for 2%. Such results indicate that respondents' support was driven by essential demands in their daily life rather than a governmental slogan. Nevertheless, the opposition to WTP was caused by unnecessary living requirements (49%), extra money (20%), and anxieties about failing to achieve expected performance (19%). About 8% of respondents were worried about unexpected consequences such as danger and inconvenience.

In Ningxia, 51% of the respondents who were willing to pay thought that convenience was their main motivation, much higher than health-related motivations (24%). About 15% of the willing respondents thought sanitation improvement could benefit environmental hygiene. In comparison, reasons for the unwillingness to pay included unnecessary living requirements (67%), doubt on expected performance (19%), extra money (5%), and unexpected consequences (5%).

In Shaanxi, 50% of the willing respondents thought sanitation improvements might benefit their health, 33% said convenience was their major concern, and 13% wanted to enjoy improved environmental hygiene. Only 2% mentioned government policy and 2% thought of other reasons as motivators. About 47% of the unwilling respondents said sanitation improvement was unnecessary, 32% thought upgrading could not improve their current sanitation sufficiently, and 12% said their most troubling concern was money.

### Influence Factors for the WTP and WTPP

Binary logistic regression was used to explore the impact of different factors on WTP and WTPP regarding sanitation improvement in Western China's rural areas. Prior to this, both the likelihood test and Hosmer–Lemeshow test were conducted to examine if the observed WTP and WTPP could match the expected WTP and WTPP (Appendix Table A1). The results indicate that there was sound goodness-of-fit (*p* > 0.1) under the logistic model and that the binary logistic regression model was applicable for subsequent analysis. Table 4 presents the variability of WTP and WTPP with demographic characteristics among the respondents.

**Table 4:** Relationships between demographic characteristics and WTP and WTPP in rural areas of the three provinces.

	**Inner Mongolia**		**Ningxia**		**Shaanxi**	
**Variable**	**WTP**	**WTPP**	**WTP**	**WTPP**	**WTP**	**WTPP**
Gender(Male)	0.59^**^ (0.37–0.93)	0.78 (0.48–1.27)	0.45 (0.13–1.51)	2.26 (0.77–6.61)	1.17 (0.82–1.66)	1.33 (0.90–1.97)
Gender(Female)	RG	RG	RG	RG	RG	RG
Health Status(healthy)	2.93^***^ (1.48–5.83)	3.44^***^ (1.73–6.85)	0.48 (0.10–2.26)	1.52 (0.38–6.05)	1.01 (0.52–1.94)	1.65 (0.78–3.50)
Health Status(Unhealthy)	RG	RG	RG	RG	RG	RG
Education(Primary school)	1.02 (0.48–2.20)	1.37 (0.61–3.08)	4.50 (0.27–75.34)	3.58 (0.40–32.20)	0.89 (0.42–1.90)	0.56 (0.24–1.32)
Education(Secondary school)	0.77 (0.38–1.57)	0.57 (0.27–1.21)	2.22 (0.34–14.30)	3.66 (0.84–16.02)	1.32 (0.67–2.58)	0.63 (0.29–1.38)
Education(High school)	1.09 (0.50–2.36)	1.44 (0.62–3.36)	2.39 (0.46–12.33)	2.46 (0.66–9.27)	1.42 (0.68–2.96)	0.73 (0.31–1.69)
Education(University and above)	RG	RG	RG	RG	RG	RG
Occupation(Service Personnel)	3.46^**^ (1.25–9.53)	2.10 (0.71–6.25)	0.18 (0.00–20.93)	0.13 (0.01–2.71)	1.01 (0.51–1.99)	2.34^**^ (1.08–5.07)
Occupation(Migrant worker)	1.63 (0.66–4.05)	1.41 (0.52–3.82)	1.82 (0.20–17.01)	0.77 (0.14–4.24)	3.48^***^ (1.5–8.08)	2.76^**^ (1.07–7.15)
Occupation(Other work)	1.63 (0.73–3.66)	1.16 (0.47–2.83)	0.80 (0.15–4.34)	1.55 (0.38–6.32)	0.74 (0.38–1.41)	0.92 (0.45–1.87)
Occupation(Farmer)	RG	RG	RG	RG	RG	RG
Family size(≤ 6)	1.37 (0.60–3.09)	1.14 (0.47–2.75)	0.87 (0.13–5.75)	2.03 (0.29–13.98)	0.80 (0.51–1.27)	0.71 (0.43–1.20)
Family size(>6)	RG	RG	RG	RG	RG	RG
Income(<1510 USD)	0.83 (0.41–1.70)	1.14 (0.53–2.45)	12.17^***^ (2.33–63.55)	13.57^***^ (1.99–92.27)	1.11 (0.69–1.78)	1.42 (0.85–2.36)
Income(1511–4532USD)	1.43 (0.80–2.56)	1.38 (0.74–2.57)	1.63 (0.25–10.69)	1.52 (0.32–7.18)	0.96 (0.59–1.54)	1.08 (0.65–1.78)
Income(>4532USD)	RG	RG	RG	RG	RG	RG

*RG, reference group.*

** *Indicates significance at the level of 0.01 and*

*** *indicates significance at the level of 0.001*.

In Inner Mongolia, the male respondents had a lower WTP level than the female respondents, about 0.59 times less. Healthy respondents were more likely to pay compared with unhealthy respondents, about 2.93 times. Meanwhile, the healthy group had a higher level of intention to provide labor, about 3.44 times that of an unhealthy group. Service personnel showed a higher WTP level, about 3.46 times that of farmers. In Ningxia, respondents with the lowest income level were more likely to pay and provide labor for improving sanitation, about 12.17 and 13.57 times that of the wealthiest group, in WTP and WTPP respectively. In Shaanxi, both service personnel and migrant workers had a higher level of WTPP, compared with farmers, about 2.34 and 2.76 times that of farmers. Moreover, in Shaanxi, the migrant workers had a higher level of WTP, about 3.48 times that of farmers.

Table 5 shows the variability of WTP and WTPP with the time living in villages, interest in notice checking, and activities of learning policies. The results indicate that in Inner Mongolia, rural residents who spent less than 3 months in the village showed stronger WTP and WTPP, about 4.03 and 4.57 times that of people who stayed in rural areas for more than 10 months. The results were different in the case of Shaanxi, where the WTP and WTPP levels of respondents who lived for less than 3 months were only 0.29 and 0.37 times that of respondents who lived for more than 10 months. In comparison, in Ningxia, the respondents who lived in villages for 4–9 months annually had a higher level of WTP (18.71 times) than those who lived in villages for 10–12 months.

**Table 5:** Relationships between personal activities and WTP and WTPP.

	**Inner Mongolia**		**Ningxia**		**Shaanxi**	
**Variable**	**WTP**	**WTPP**	**WTP**	**WTPP**	**WTP**	**WTPP**
Time spent in village(Less than 3 months)	4.03^***^ (1.79–9.09)	4.57^***^ (1.85–11.26)	0.22 (0.03–1.57)	0.39 (0.09–1.84)	0.29^***^ (0.13–0.65)	0.37^**^ (0.16–0.82)
Time spent in village(4–9 months)	2.17 (0.85–5.53)	2.47 (0.88–6.88)	18.71^**^ (1.52–230.25)	6.08 (0.38–98.35)	1.04 (0.62–1.72)	1.74 (0.91–3.33)
Time spent in village(10–12 months)	RG	RG	RG	RG	RG	RG
Notices checking(Least frequent)	0.18^**^ (0.05–0.73)	0.18^**^ (0.04–0.91)	0.02^***^ (0.00–0.19)	0.06^**^ (0.01–0.73)	0.25^***^ (0.09–0.68)	0.70 (0.24–2.09)
Notices checking(Less frequent)	0.17^**^ (0.04–0.70)	0.13^**^ (0.03–0.65)	0.10^**^ (0.01–0.82)	0.03^***^ (0.00–0.36)	0.48 (0.17–1.34)	0.72 (0.23–2.28)
Notices checking(Moderately frequent)	0.39 (0.09–1.63)	0.33 (0.06–1.72)	0.03^***^ (0.00–0.36)	0.24 (0.02–3.47)	0.59 (0.22–1.60)	1.28 (0.42–3.90)
Notices checking(Frequent)	0.33 (0.07–1.46)	0.32 (0.06–1.77)	0.15 (0.01–1.69)	0.13 (0.01–1.95)	0.60 (0.18–2.02)	1.05 (0.26–4.25)
Notices checking(Most frequent)	RG	RG	RG	RG	RG	RG
Learning policy (No)	0.68 (0.42–1.10)	0.85 (0.51–1.42)	0.18^***^ (0.05–0.65)	0.93 (0.34–2.56)	0.47^***^ (0.29–0.75)	0.39^***^ (0.21–0.72)
Learning policy (Yes)	RG	RG	RG	RG	RG	RG

*RG, reference group.*

** *Indicates significance at the level of 0.01 and*

*** *indicates significance at the level of 0.001*.

The respondents (least frequently checked the notices) showed less interest in paying and participating in all three provinces (0.02–0.48 times) than the respondents (most frequently checked the notices), apart from the case of WTPP in Shaanxi. In both Inner Mongolia and Ningxia, respondents who checked the notices less frequently also had a lower level (0.03–0.17 times) of WTP and WTPP than the respondents who checked the notices most frequently. The rural respondents who did not learn the policy had a lower level of WTP and WTPP in Shaanxi than the respondents who learned, about 0.47 and 0.39 times, respectively. In Ningxia, the WTP level among the respondents who did not learn was also lower, about 0.18 times that of respondents who learned.

Table 6 shows the variability of WTP and WTPP with sanitation services and infrastructure in villages. In Inner Mongolia, respondents who did not suffer difficulties in getting safe drinking water showed higher interests in paying and participating, about 2.23 and 3.18 times that of suffering difficulties. In Inner Mongolia, respondents who did not suffer difficulties in getting safe drinking water showed higher interests in upgrading sanitation than respondents who suffered difficulties in getting safe drinking water. Sanitation quality could also affect respondents' WTP and WTPP. Respondents could have a higher level of WTP and WTPP if no sanitation improvement was taken in the past 10 years, especially in Ningxia, reaching 30.42 and 67.09 times for WTPP and WTP, respectively. Moreover, a 5-year gap led to a higher level of WTPP (4.24 times) in Ningxia and WTP (1.72 times) in Shaanxi. However, the willingness of residents was not affected by whether the sanitation was connected with toilet.

**Table 6:** Relationships between sanitation service and infrastructure and WTP and WTPP.

	**Inner Mongolia**		**Ningxia**		**Shaanxi**	
**Variable**	**WTP**	**WTPP**	**WTP**	**WTPP**	**WTP**	**WTPP**
Difficulties of water (No)	2.23^**^ (1.19–4.20)	3.18^***^ (1.53–6.63)	4.32 (0.67–27.95)	0.65 (0.12–3.69)	1.59^**^ (1.01–2.50)	1.68 (0.98–2.86)
Difficulties of water (Yes)	RG	RG	RG	RG	RG	RG
Time of sanitation upgraded (Above10 years)	1.89^**^ (1.09–3.26)	1.78^**^ (1.01–3.14)	67.09^***^ (10.36–434.62)	30.42^***^ (6.25–148.18)	2.39^***^ (1.47–3.89)	2.68^***^ (1.45–4.94)
Time of sanitation upgraded (5–10 years)	1.14 (0.57–2.27)	1.24 (0.6–2.57)	4.56 (0.89–23.47)	4.24^**^ (1.20–14.97)	1.72^**^ (1.12–2.62)	1.09 (0.68–1.75)
Time of sanitation upgraded (Less than 5 years)	RG	RG	RG	RG	RG	RG
Connected toilets (No)	1.19 (0.41–3.44)	1.12 (0.33–3.79)	0.20 (0.01–2.76)	0.21 (0.03–1.7)	0.55 (0.29–1.05)	0.81 (0.4–1.65)
Connected toilets (Yes)	RG	RG	RG	RG	RG	RG

*RG, reference group.*

** *Indicates significance at the level of 0.01 and*

*** *indicates significance at the level of 0.001*.

## Discussion

This article explored rural residents' support for sanitation improvement, economically and physically, in rural areas of Inner Mongolia, Ningxia, and Shaanxi. The study used a binary logit model to analyze the explanatory factors for WTP and WTPP variations. This study generated not only new findings, presented in Section Results, but also have theoretical and practical implications.

### Exploratory Factors of Willingness to Pay and Participate

#### Gender Difference in Willingness

Gender was a factor that affected WTP in Inner Mongolia, and women had a higher level of WTP for sanitation improvement. The results are in tandem with previous studies that demonstrated eco-friendly actions are impacted by gender (54, 55). In other words, women are more positive regarding pro-environmental behavior. Moreover, the higher level of WTP among women might be relevant to more household works performed by women rather than men in the study areas, implying that upgrading sanitation could result in more benefits to women than men. In comparison, gender did not affect WTPP in Inner Mongolia, which might be because of the great requirement in physical strength, making both men and women reluctant to contribute. Moreover, there are insignificant differences in the WTP and WTPP with gender in both Shaanxi and Ningxia. This result may be because of the higher social status of female Mongols (56). Women with higher social status may strengthen their bargaining power in the household and own greater participation in community activities (57). Inner Mongolia is the Mongolian Autonomous Region. Thus, females in Inner Mongolia show more willingness to express their opinions about improving sanitation. In other provinces, this willingness may disappear when the social status of female declines. Thus, we observed no significant connection between gender and willingness in other provinces.

#### Health Concerns Behind the Choice

Health status could arouse respondents' WTP, but only in Inner Mongolia. The healthy group could have a higher level of WTP and WTPP than the unhealthy group, which is consistent with the view of Khan et al. and Wang et al. (58, 59). They stated that health status could significantly influence respondents' attitudes. The result was understandable, because illnesses could result in a financial burden on unhealthy respondents, and at the same time, decrease their ability to provide labor to improve sanitation. However, significant impacts of health were not observed in both Ningxia and Shaanxi; this might be because Ningxia and Shaanxi had an integrated urban-rural household health insurance system (60, 61). As a result, the rural residents could obtain higher compensation payouts. In comparison, the urban and rural household health insurance system was not fully integrated into Inner Mongolia, which meant that unhealthy rural residents had to shoulder a large part of expenses. However, the results should be further explored.

#### Knowledge Differences in Willingness

Education in relevant subjects has been considered a factor that affects respondents' attitudes (62). The results of our study indicated that respondents who learned about sanitation policies had a higher level of WTP and WTPP than rural residents who did not in Shaanxi (Table 6). The results were consistent with previous studies that knowledge is a driver to respondents' attitudes (63). Furthermore, the respondents who learned about sanitation policies had a high level of WTP in Ningxia, indicating that respondents' attitude was affected. Such results imply the significance of policy propaganda that could not only convey the policy but also deliver basic knowledge of sanitation improvement (64). However, sanitation policy did not affect respondents' WTP in Inner Mongolia, and WTPP in both Inner Mongolia and Ningxia, where the cause might interact with other factors, and more studies should be conducted to explore this.

#### Willingness Among Occupations

Yang et al. argued that occupations could influence respondents' willingness (65). In this study, we found that service personnel in Inner Mongolia and migrant workers in Shaanxi were more active in WTP compared with farmers. Service personnel and migrant workers were active in WTPP compared with farmers in Shaanxi. Such results may be because the service personnel and migrant workers had different lifestyles than the farmers. The farmers spend lots of time in the village, while the service personnel and migrant workers could have higher levels of living requirements after getting in touch with high quality of life. However, the occupation did not have a significant impact on WTP and WTPP in Ningxia. This result may be relevant to the reluctance of rural residents in Shaanxi and Inner Mongolia to migrate, and their willingness to stay in their hometowns; even if they must leave, they prefer to stay close to their hometown. Accordingly, the migrant workers and service personnel in Shaanxi and Inner Mongolia would be more likely to settle in towns and cities near their villages and maintain a close connection with the villages.

### Implications for Sanitation Improvement

#### Living Demands of Rural Residence

The WTP and WTPP were significant and related to the time spent in their villages. The respondents who spent less than 3 months in villages showed a higher level of WTP and WTPP in Inner Mongolia, less WTP in Ningxia, and a lower level of WTP and WTPP in Shaanxi. The respondents who spent 4–9 months in villages had a higher level of WTP in Ningxia. This result complies with early studies that indicate that length of stay could have a statistically significant impact on respondents' WTP (66). Overall, the results indicate that respondents who were not fully living in villages would be more active. Such results might be because once people live in cities and towns with qualified sanitation services and infrastructure, they would have a higher level of quality of life. As a result, sanitation improvement became essential for them (47). Tuan et al. argue that respondents whose lifestyles were more dependent on the subject were more willing to provide contributions (67). For some rural residents, a long time of living in environments without sanitation services and infrastructure made them used to the quality of living, even though the quality was low. As a result, upgrading sanitation would be less attractive for these rural residents. From this perspective, the government propaganda pilot projects to enable rural residents to understand sanitation performance. Basic knowledge of sanitation benefits and relevant policies can be delivered to rural residents to overcome the barriers of old ideas and lifestyles.

#### Insufficient Concerns of Rural Government Notices

The frequency of notice-checking was associated with respondents' WTP in all provinces and the WTPP in Inner Mongolia and Ningxia. Such results indicate that an improved concern of notices of village government could allow rural residents to understand the latest policies and information on sanitation improvement. There might be an improvement in the likelihood of trusting village government and the expected performance. This finding is identical to the suggestion of Liu et al., who stated that altruistic individuals were observed to be more positive regarding eco-friendly technology (68). However, notice-checking did not have a significant effect on WTPP, which might be because of the interactions among other factors in Shaanxi. Nevertheless, the positive relationships in other provinces were the evidence for the importance of their notices. Accordingly, village government notices should be presented in a positive way, where the notices could be not only shown on a bulletin board and in village broadcasting stations but also can be delivered on a website and social networking software or application to break the barriers constraining information dissemination.

#### Failure and Disorder of Old Sanitation

The latest upgrading time was another factor strongly affecting rural residents' WTP and WTPP. The longer the sanitation was not upgraded, the more respondents were willing to pay. This result is identical to a related study conducted by Kumie et al. who argued that insufficient demand would be the main barrier to improvement (69). The fact that the longer sanitation was not upgraded, means that the old sanitation services and infrastructure cannot meet the basic requirements because of failure and disorder. At that time, the payment for the repair, replacement, and maintenance increased, and the repair fee would increase with time. Moreover, with improvement in living quality, old sanitation services and infrastructure could be out of date to meet emerging requirements, especially satisfaction, which could be a driver to improvement (70–72).

#### Neglected Water Quality

Rural residents who lived in arid regions or areas with poor-quality water resources could be less active in upgrading sanitation than respondents who could easily get safe drinking water in Inner Mongolia and Shaanxi. The findings of relevant studies are in accordance with our results, as Chatterjee et al. and Lera-López et al. argue that rural respondents would show a positive attitude toward upgrading sanitation to upgrade water quality (73, 74). Based on our second-round field work, it was found that safe drinking water was obtained from nearby areas through the local government. In some arid regions, rural residents even need to build a cellar to collect and contain rainwater as their drinking water (75). Some rural residents who suffered poor water quality and did not get safe drinking water from the government, however, chose to purchase bottled water. This means that rural residents who could not easily get safe drinking water did not drink the water in their villages, and that they were less impacted by insanitary water locally. In general, the respondents' (with no difficulties in water availability) active attitudes toward WTP and WTPP were an action to protect the water resources they were dependent on.

### Limitations

This study had a few limitations. First, it used a questionnaire structure; thus, other factors might impact rural residents' WTP and WTPP in improving sanitation that was not included in the questionnaire. Second, the limited number of respondents might cause bias and variability. Although the options on the questionnaire were revised based on the opinions from three focus group discussions, using a validated instrument to confirm our results in further research is required.

## Conclusions

The support of rural residents is indispensable to sanitation improvements in rural areas. This study investigated the WTP and WTPP of rural residents regarding sanitation improvement. The results indicate that the respondents of Inner Mongolia had the highest level of WTP and the lowest level of WTPP. In comparison, the respondents in Ningxia had the lowest level of WTP, and those in Shaanxi had the highest level of WTPP. Moreover, there was a mean fixed payment of USD 55.75 in Inner Mongolia, USD 59.82 in Ningxia, and USD 45.20 in Shaanxi per rural household.

It is noteworthy to notice that time spent in the village had a statistically significant association with willingness. The respondents who actively check notices from the government were more positive about promoting sanitation. The latest upgrading time has a significant impact on rural residents' WTP and WTPP. The respondents who learned about sanitation policies would have greater willingness for sanitation improvements in Inner Mongolia and Ningxia. Service personnel in Inner Mongolia and Migrant workers in Shaanxi would show more interest in upgrading sanitation compared with farmers. In Inner Mongolia and Shaanxi, the rural residents who suffered from difficulty in getting safe drinking water could have a lower level of willingness to upgrade sanitation than other rural residents. In Inner Mongolia, females showed a more positive attitude to improve sanitation. The healthy group showed more WTP and WTPP than the unhealthy group, but only in Inner Mongolia. The results were complicatedly affected by demographic characteristics, personal activities, and sanitation services and infrastructure in villages. However, the impacts of such factors varied significantly with provinces, indicating that the policy and actions for improving people's willingness should be tailored to a local context. Overall, the survey could serve as a useful reference for conducting sanitation studies and creating rural sanitation policies.

## Data Availability Statement

The raw data supporting the conclusions of this article will be made available by the authors, without undue reservation.

## Ethics Statement

Ethical approval was not provided for this study on human participants because there is no Ethical Committee in our University. However, we highly respect the privacy and rights of the possible respondents. The patients/participants provided their written informed consent to participate in this study.

## Author Contributions

SW: formal analysis, investigation, methodology, software, validation, visualization, roles, writing—original draft, and writing—review and editing. B-JH: data curation, formal analysis, funding acquisition, methodology, project administration, resources, visualization, roles, writing—original draft, and writing—review and editing. YZ: conceptualization, funding acquisition, project administration, resources, and writing-review and editing.

## Funding

Project Nos. 2021CDJQY-004 and 2021CDJQY-023 supported by the Fundamental Research Funds for the Central Universities.

## Conflict of Interest

The authors declare that the research was conducted in the absence of any commercial or financial relationships that could be construed as a potential conflict of interest.

## Publisher's Note

All claims expressed in this article are solely those of the authors and do not necessarily represent those of their affiliated organizations, or those of the publisher, the editors and the reviewers. Any product that may be evaluated in this article, or claim that may be made by its manufacturer, is not guaranteed or endorsed by the publisher.

## Appendix

Table A1 presents the results of the likelihood test and Hosmer–Lemeshow test. The Nagelkerke R square is the revised Cox and Snell R Square. Coefficients of the Nagelkerke R square and Cox and Snell R square indicated that all the groups could match the expected results well (76). The Hosmer–Lemeshow test provided an analysis of the overall fit of the models (77). The Chi-square coefficients of the Hosmer–Lemeshow test were all greater than 0.05, so the expected results could be fit well.

**TABLE A1:** Model fitting information.

**Model**		**Likelihood test**			**Hosmer-Lemeshow Test**		
		**Log likelihood**	**Cox & snell R square**	**Nagelkerke R square**	**Chi-square**	**df**	**Sig**
**Inner Mongolia** **(*N* = 441)**	WTP	507.73	0.19	0.26	5.11	8	0.75
	WTPP	460.71	0.21	0.29	4.65	8	0.79
**Ningxia** **(*N* = 136)**	WTP	92.81	0.42	0.59	4.45	8	0.81
	WTPP	116.14	0.34	0.47	4.49	8	0.81
**Shaanxi** **(*N* = 671)**	WTP	789.27	0.19	0.25	4.5	8	0.81
	WTPP	665.57	0.13	0.19	10.2	8	0.25
